# Seasonal autoregressive integrated moving average (SARIMA) time-series model for milk production forecasting in pasture-based dairy cows in the Andean highlands

**DOI:** 10.1371/journal.pone.0288849

**Published:** 2023-11-16

**Authors:** Uri H. Perez-Guerra, Rassiel Macedo, Yan P. Manrique, Eloy A. Condori, Henry I. Gonzáles, Eliseo Fernández, Natalio Luque, Manuel G. Pérez-Durand, Manuel García-Herreros

**Affiliations:** 1 Facultad de Medicina Veterinaria y Zootecnia, Universidad Nacional del Altiplano, Puno, Peru; 2 Facultad de Ciencias Agrarias, Universidad Nacional San Antonio Abad del Cusco, Cusco, Peru; 3 Instituto Nacional de Investigação Agrária e Veterinária, I. P. (INIAV, I.P.), Santarém, Portugal; Rajarata University of Sri Lanka Faculty of Medicine and Allied Sciences, SRI LANKA

## Abstract

Milk production in the Andean highlands is variable over space and time. This variability is related to fluctuating environmental factors such as rainfall season which directly influence the availability of livestock feeding resources. The main aim of this study was to develop a time-series model to forecast milk production in a mountainous geographical area by analysing the dynamics of milk records thorough the year. The study was carried out in the Andean highlands, using time–series models of monthly milk records collected routinely from dairy cows maintained in a controlled experimental farm over a 9-year period (2008–2016). Several statistical forecasting models were compared. The Mean Absolute Error (MAE), Root Mean Square Error (RMSE), and Mean Absolute Percent Error (MAPE) were used as selection criteria to compare models. A relation between monthly milk records and the season of the year was modelled using seasonal autoregressive integrated moving average (SARIMA) methods to explore temporal redundancy (trends and periodicity). According to white noise residual test (Q = 13.951 and p = 0.052), Akaike Information Criterion and MAE, MAPE, and RMSE values, the SARIMA (1, 0, 0) x (2, 0, 0)_12_ time-series model resulted slightly better forecasting model compared to others. In conclusion, time-series models were promising, simple and useful tools for producing reasonably reliable forecasts of milk production thorough the year in the Andean highlands. The forecasting potential of the different models were similar and they could be used indistinctly to forecast the milk production seasonal fluctuations. However, the SARIMA model performed the best good predictive capacity minimizing the prediction interval error. Thus, a useful effective strategy has been developed by using time-series models to monitor milk production and alleviate production drops due to seasonal factors in the Andean highlands.

## Introduction

Human food supply and health control continue to be important problems for the growing population [1, 2]. This is especially important in the communities located in the Andean highlands because of the limited resources and seasonal food availability [3, 4]. In the Andean highlands the animal-source food supply is critical because the populations are exposed to factors that strongly influence the animal production systems such as unstable environmental climatic conditions [5, 6]. Animal production monitoring is essential for planning and implementing efficient control of productivity [7, 8]. The environmental heterogeneity in the Andean highlands such as rainfall and temperature play a major role on pastoral production systems [9]. The extreme climatic conditions (alternate dry and rainy seasons) determine the lack of pasture availability for livestock, so there is a need of more effective control measures to be implemented due to nutrition is the most important factor that affects the animal productivity [10, 11]. Dairy production is variable over space and time and this variability is related to environmental climatic changes and poor knowledge about appropriate management and livestock feeding [12, 13]. Thus, the high milk productivity during the rainy season, when pasture availability peaks, contrasts with the low productivity during the dry season. Moreover, due to the lack of data availability from dairy production systems (poor record keeping) and the variable effects of the climatic factors, there is a growing need for methods that allow forecasting for early milk production detection in areas of unstable production, such as the Andean highlands. The lack of existing records due to technical difficulties and management-derived factors in obtaining farm-derived data from field has a severe impact on livestock productivity [14]. This fact determines one the most important limiting factors that affect the livestock productivity and socio-economic performance [15, 16]. Forecasting milk production could provide interesting information for dairy producers to establish a plan to mitigate production drops during the non-favourable dry season. Despite this strong relationship, seasonal effects have not been included in retrospective forecast models for milk production in the Andean highlands. Several models have been designed for forecasting dairy production [17]; however, despite the strong relationship between milk production and climatic factors, seasonal environmental effects have rarely been included in milk production models, probably because of technical difficulties in obtaining environmental data on these environments [17]. Current advances in forecasting models and spatial-temporal analysis facilitate the development of novel livestock-related predictions by using different approaches. The autoregressive integrated and moving average (ARIMA) models have shown their usefulness in the monitoring of dispersion patterns of diseases [2, 18–20]. These models included trend and seasonal patterns as covariates and have the ability to cope with stochastic dependence of consecutive data. ARIMA models make use of previous observations (lineal combinations of previous data and its residuals) to forecast of future values using lag parameter values [21]. ARIMA models consist of some subset models such as simple and multiple regression together with moving averages. Time-series can also be used to study the association among several variables that change over time and influence each other. Multiple time-series would have to be modelled at more refined spatial scales in order to get well-fitting models [21]. ARIMA models provide good information for testing control measures, and risk factors [22, 23]. However, there is limited information about ARIMA model application for forecasting milk production patterns on different climatic conditions. Patterns of milk production could be modelled for cattle using time-series analyses using data daily aggregated at the local level which could be fitted using seasonal ARIMA (SARIMA) models. However, there is limited information from studies in highland areas evaluating the methods that would allow forecasting milk production ahead.

The hypothesis stated in the present research is that milk production in a particular month can be forecasted by seasonality. The objective of this work was to study the relation among months/season to develop a forecasting model that can prognosticate the milk production with reasonable reliability in pasture-based dairy cows in the Andean highlands. Thus, the aim was to provide a temporal model of milk production based on time-series models adapted to field data from the Andean Highlands as well as to analyse the incidence fluctuations of milk production over a decade assessing the presence or absence of cyclic tendencies.

## Materials and methods

### Ethical statement

This research was performed in strict accordance with the recommendations in the legal framework (Animal Welfare Law) for all Peruvian Public and Private Laboratories and Higher Education Institutions. " The study was conducted according to the guidelines of the Declaration of Helsinki and following the Code of Ethics for animal experiments as reflected in the ARRIVE guidelines available at http://www.nc3rs.org.uk/ARRIVEchecklist (Accessed on 7 July 2020). This study was approved by the Bioethics Committee for the use of experimental animals at the Universidad Nacional del Altiplano—Puno—Perú (Approval Date: 30 June 2020, Code Number: CMTA-019-UNA-CE)".

### Study area

**Fig 1:**
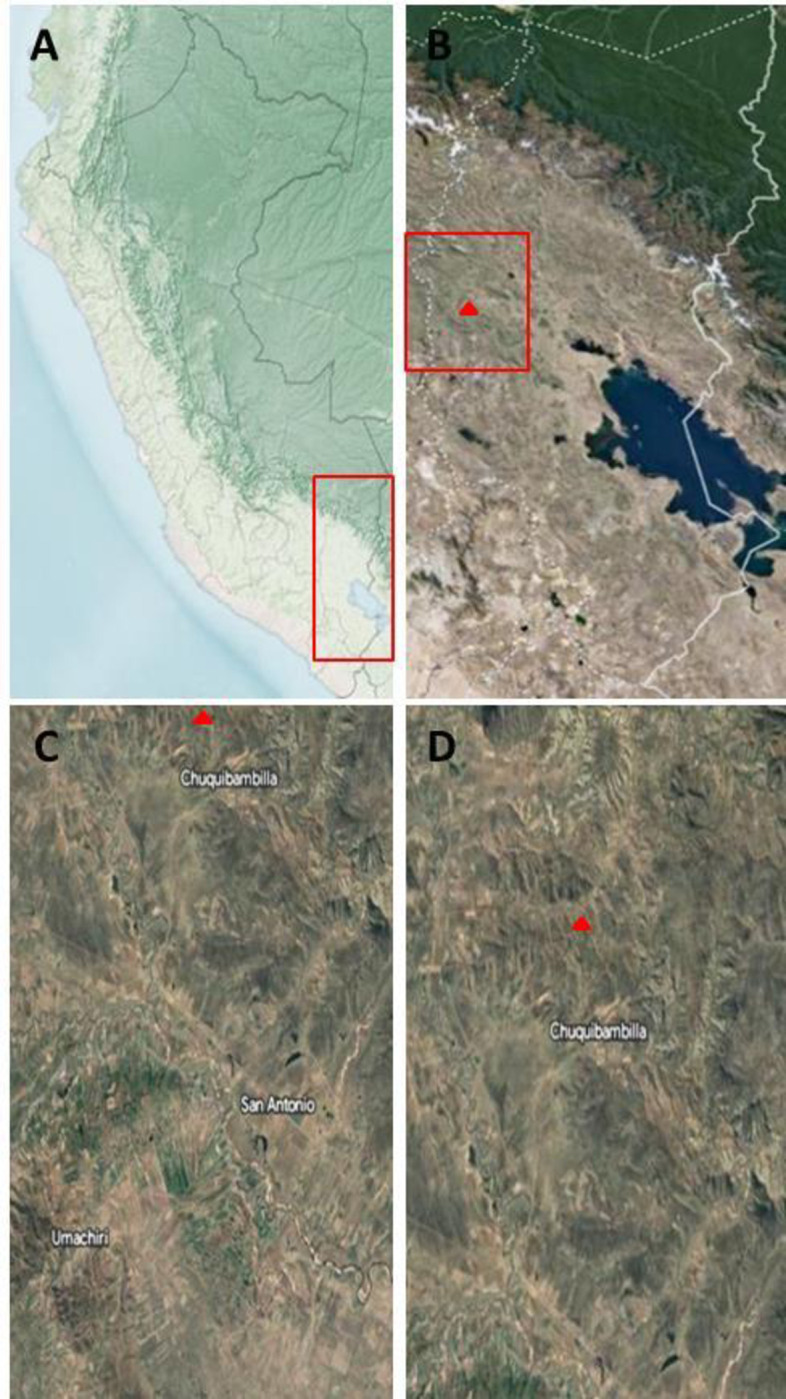
Study area location in the Andean Highlands of Perú (country, department, province, district, and area) where data collection was performed including seasonal data and milk production performance. A) Country: Perú; Departament: Puno (red square). B) Departament: Puno; Province: Melgar (red square); District: Umachiri (red tringle). C) District: Umachiri; Area: Chuquimbabilla (red triangle). D) Andean highlands study area in Chuquibambilla Experimental Research Centre (red triangle) where the milk production data were collected (~ 4,000 m.a.s.l.; Latitude: 14° 44′ 40.38″ N; Longitude: 70° 41′ 55.28″ W). This figure is not identical to the original image and is therefore for illustrative purposes only. USGS EROS (Earth Resources Observatory and Science (EROS) Center) (public domain): https://earthexplorer.usgs.gov/.

Milking records were monitored daily by a field study (2008 to 2016) from the experimental cattle herd of the National University of the Altiplano (Puno, Peru) located in the central Andean mountain range at an altitude of ~ 4,000 m.a.s.l. (Latitude: 14° 44′ 40.38″ N; Longitude: 70° 41′ 55.28″) (Fig 1). The Koppen climate classification is Cwb (subtropical highland climate) with mean annual temperature of ~10 ± 3.8°C and a relative humidity of ~85%. This herd is representative of the small farming activity performed in this region. The study included a herd composed by 66±9 Brown Swiss breed lactating cows of similar characteristics (BW: 450± 50 kg; BCS: 2.5–3.5) maintained in a natural extensive pasture-based system (grazing from 8:00 a.m.-5:00 p.m.) and water ad libitum. All cows were milked twice a day (6:00 a.m.– 6:00 p.m.). The individual milking records were obtained daily by means of the scale of the milking system in order to have longitudinal information that allows us to perform the present study.

### Data processing

The seasonal autoregressive integrated by moving average (SARIMA) model was defined based on the Box and Jenkins methodology for stationary time-series [24]. The analysis of the general class of SARIMA (p, d, q) and (P, D, Q) structures was carried out, where ´p´ was the order of the non-seasonal autoregressive polynomial, (´P´ was the order of the seasonal autoregressive polynomial); d was the order of non-seasonal differentiation (´D´ was the order of seasonal differentiation), and ´q´ was the order of the non-seasonal moving average polynomial (´Q´ was the order of the seasonal moving average polynomial). This analysis needs variance and means stationary data. The linear model consisted, as stated before, of three key parameters of ´p´, ´d´, and ´q´ where ´p´ indicates the auto-regressive (AR) part of the model. Moreover, ´p´ incorporates the effect of lag value, and ´d´ represents the number of non-seasonal differences needed for model stationary. The moving average (MA) was represented with ´q´ which was the number of lagged forecast errors in the prediction equation. The identification of SARIMA models was divided in three parts: a) identification: consisted on the determination of filters to apply to the series to come down to a stationary process and the choice of the orders ´p´ and ´q´ of the autoregressive and moving-average operators; b) estimation: consisted on the selection of values for the coefficients of the polynomials ´p´ and ´q´; and finally, c) diagnostic checking: consisted on the validation tests which verify especially that the process (w; t E Z) was really a white noise process.

### Modelling procedure

In the present study the steps of the modelling procedure were carried out as follows: a) the data matrix was plotted and unusual observations were identified (if any); b) the data matrix was transformed to stabilize the variance (if necessary); c) the data stationarity was checked (if they were non-stationary the data were adjusted till be stationary); d) autocorrelation function was performed to disclose the most appropriate SARIMA model (e.g., p, d, 0 or 0, d, q); e) the selected model was executed and adjusted for a better fitting by using AICs; f) the residuals were tested from the selected model by plotting the autocorrelation function together with the Portmanteau test of the residuals to compare with the white noise time series. If the residuals didn´t fit with the white noise then the model was modified; and finally, g) the forecasts were calculated.

### Performing the SARIMA model

First the aim was to apply the Bartlett test for variance stationary analysis. After that, the Augmented Dickey- Fuller (ADF) unit-root test for the mean stationery was performed. Moreover, the square root transformation was used for variance stationary because it doesn’t need the regular difference to remove the trend. A variation based on the Hyndman & Khandakar [25] algorithm combining unit root tests, minimizing the Akaike Information Criterion with correction (AICc), and minimizing the maximum likelihood estimation (MLE) were performed to obtain the SARIMA model applied to monthly milk production records in the Andean highlands. Variations on the algorithm were carried out when required.

First of all a default procedure was performed to search for approximations; however, this initial procedure it does not allow for the constant c (unless d = 0), neither allow the estimated model to be applied to new data. Then, the Portmanteau tests of residuals were performed due to there are more accurate, especially if the degrees of freedom of the test statistic are adjusted with regard to the number of parameters in the model. Therefore, ℓ – K degrees of freedom in the test were used being K the number of AR and MA parameters in the model. Autocorrelation functions (ACFs) and partial autocorrelation functions (PACFs) were identified and examining to see which of the potential three patterns were present in the data. After selecting the appropriate SARIMA model its coefficients were estimated and, finally, a diagnosis was carried out.

The residuals were analysed in order to prove whether the model that fitted our data was adequate. Ljung-Box test, Shapiro-Wilk and Kolmogorov-Smirnov tests were used to check independence and normality of residuals (p-value>0.05 was considered, accepting the hypothesis of independence or normality). The diagnosis was carried out performing an analysis of residuals and verification tests (Ljung–Box) to assess whether the suggested model is suitable for predictions using the functions ‘tsdiag ()’, ‘Box.test ()’, and ‘shapiro.test ()’, which are native to the R Studio software (v. 4.0.3). Model performance was estimated by calculating the amount of the original series variance that was explained by the model. This is analogous to the coefficient of determination (R^2^) of a linear model. A log transformation and differencing of the raw series to achieve stationarity were performed. Identification of the order of differencing and estimating the variance of the series was carried out using ACF plots. A seasonal autoregressive integrated moving-average (SARIMA) process was fitted using the methods and diagnostics described previously. The predictive ability of the model was evaluated by two methods: (1) plotting the forecast and its 90% tolerance intervals and comparing it with the observed data; and (2) calculating the root-mean squared percentage error criterion. Thus, for the non-seasonal models the equation K = p + q was considered.

The next step was to perform a time-series decomposition to split it into several components, each one each representing a specific pattern category. Decomposition methods were used to identify and isolate each of the variational components that were present in the series (trend, seasonality, heteroskedasticity, etc.). Stationarity was analysed and once the time-series was “stabilized” using appropriate transformations a study of the presence of regularities in the series was made in order to identify a possible SARIMA model. Each time series, Yt, was decomposed into two parts, trend part and error part, Yt – m(t) = єt where m(t) represents the trend and εt the error. For calculating the error, the seasonal and irregular parts of the time t were considered. Once the two parts of the series were separated, trend and error, the SARIMA methodology was applied to the random part. Considering two time-series (e.g., Y1, Y2) the distance between both series was defined (where m1, m2 are the trends in each series, θ1, θ2 the set of parameters of the SARIMA model for the random part and ω(t) a measure of weight. In this case, ω(t) = 1, all time-series have the same weight).

As the SARIMA models for the random part were practically identical, in order to determine the distance between milk production series, the distance between trends was calculated. Once this distance was defined a multivariate analysis (cluster) method was applied in order to obtain homogeneous groups. Before applying the distance, the trend data were standardized to mean = 0 and variance = 1. Three types of time-series components were considered: trend, seasonality/autocorrelation, and cycle. Time-series methods describe these components. The exploration of trends facilitates the control of the analysis process and the seasonality or other temporal autocorrelation identification.

To describe the trend, the monthly milk production record was presented as a time-series graph. The presence of trend was formally tested using a bootstrapped Spearman test. A combination of the trend and cycle into a single trend-cycle component was achieved for simplicity. Thus, the time-series comprises three components: trend-cycle, seasonal, and a remainder. Thereafter `Seasonal and Trend decomposition using Loess´ (STL) method for decomposing time-series was executed [26] due to this method can manage any type of seasonality and permits to change the seasonal component over time. For all time-series plots loess smoothing splines were applied to the raw time-series to emphasize the major features while reducing distraction from random variation. The data were rendered stationary to provide a degree of replication within the series facilitating further statistical analysis. The log of the proportion of samples positive was performed to stabilize the variance and then detrended the series by fitting a priori a second-order polynomial to stabilize the mean. These transformations were performed on the overall time series allowing a more detailed examination of spatiotemporal variation. There are some advantages of this method. For example, the rate of change and the smoothness of the trend can be controlled by the user. Another advantage of STL method was the robustness against outliers which could affect the reminder but no the estimates of trend-cycle and seasonal components.

The time-series was represented by the combination of the trend-cycle, seasonality, and reminder/error components and were described as follows: Zt = Tt + St + Et where Zt is the data observed in period t, Tt is the trend-cycle component in period t, St is the seasonal component in period t, and finally Et represents the part not captured in the model in period t, called error or random residual. The time-series (Zt) of milk production records was decomposed using the function ‘decompose ()’, native to the R Studio software so that its three components could be viewed individually. The trend-cycle component (Tt) was analysed by linear regression using the function ‘lm ()’, native to the R Studio software. The functions ‘efp ()’ and ‘sctest ()’, belonging to the statistical package ‘strucchange’ were used to verify the existence of structural breaks in the process of the time-series under study by using the statistical method ‘OLS-CUSUM’. The occurrence of milk production cycles was assessed using the function ‘filter ()’. The existence of the seasonal component (St) in the time-series was verified using the functions ‘qs ()’, ‘sesas ()’, and ‘series ()’, belonging to the statistical package ‘seasonal’ of the program ‘X-13 ARIMASEATS’. The next step was to perform an exponential smoothing procedure (e.g., Holt-Winters additive method) to capture seasonality. This forecasting method considers weighted averages of past observations, being the more recent the higher the associated weight and it was convenient when the seasonal variations are constant through the series. The method needs to recognize trend and seasonal components of the time-series and the way in which these components enter the smoothing method. The seasonal component is expressed in absolute terms, and in the level equation the series is adjusted by subtracting the seasonal component. The level equation shows a weighted average between the seasonally adjusted observation (yt – st – m) and the non-seasonal forecast (ℓt – 1 + bt – 1) for time t. A final forecast was carried out by applying the function ‘forecast ()’ of the statistical package forecast was used to forecast milk production.

The accuracy of the SARIMA predictive model in future observations was assessed by the cross-validation of Holdout, aiming to assess the generalization capacity of the model. For this, the function ‘window ()’, native to the R software [27], was used. In this case, the data from 2008 to 2016 were used as training and the data from 2017 were used as a test, considering the value of Theil’s U-statistic, whose result must be <1 for the model to have a good predictive capacity. The reliability criteria for the forecast consisted in verifying that: a) the difference between the predicted and observed value was white noise or a normal random variable with a mean = 0 and standard deviation = 1. The randomness of the difference was tested based on the trend analysis of the data and normality was tested using the Kolmogorov-Smirnov test; b) the differences did not exceed the limits of the 95% confidence interval > 5%; values falling outside the confidence interval were counted using the difference (y axis) with respect to the observed rate (x axis); and c) the differences did not tend to increase or decrease when the observed milk record increases. Therefore, the precision of the forecast was not dependent on the magnitude of the record. The model was tested using the sub-series of milk records separated and its reliability was tested by comparing each predicted record with that observed for the corresponding month. To test this, the correlation between the difference and the observed record was estimated using the Pearson linear correlation coefficient. Trend analysis was used to test the statistical significance (slope of the trend) of the difference with respect to the observed rate.

Finally, the Mean Absolute Error (MAE), Root Mean Square Error (RMSE), and Mean Absolute Percent Error (MAPE) were calculated for the evaluation of the model accuracy. Data processing was performed using the R statistical package [27] to evaluate the seasonal effect on milk production records. A 2-tailed significance level of 0.05 was established for all tests.

## Results

### Seasonal pattern of milk production

**Fig 2:**
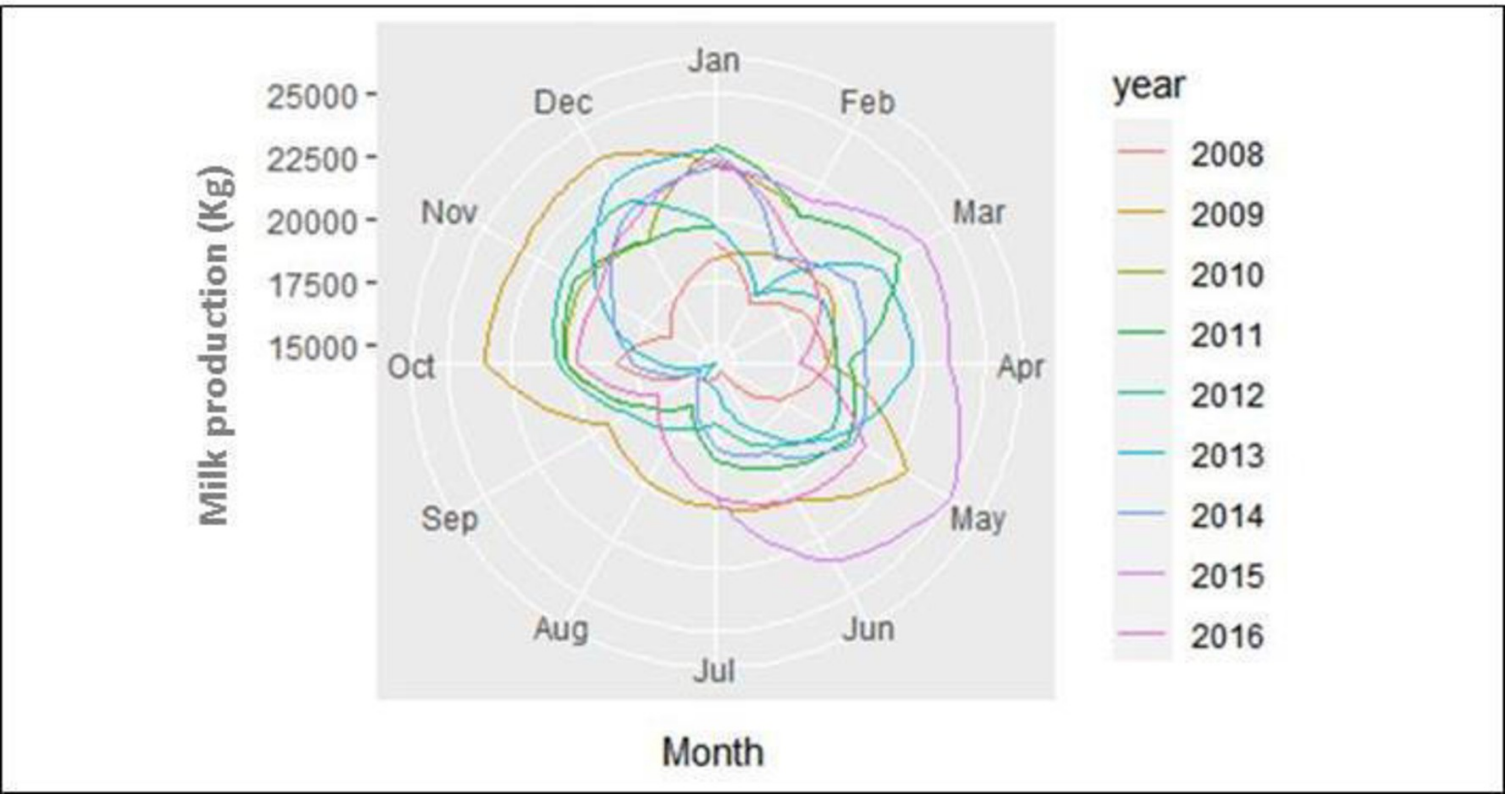
Seasonal pattern of milk production (kg) by month in dairy cows maintained in the Andean highlands. The seasonal plot clearly shows a direct influence of climate variations such as the rainy season (October ‐ March) where the highest milk production is observed and the dry season (April ‐ September) where in most years there is a significant decrease in milk production.

The seasonal pattern of milk production was significantly explained by the forecast model; however, before starting a time-series analysis, it is necessary to identify the stationarity of the data series. The exploration of milk production records for 2008–2016 shows a slight decreasing trend (2008–2014), and suggests a seasonal fluctuation in the series (R^2^ = 0.63), with a 6-month period for increased milk production (rainy season; October-March) and a 6-month period for decreasing milk production (dry season; April-September) indicating a rhythmic oscillation for both variables (Fig 2).

**Fig 3:**
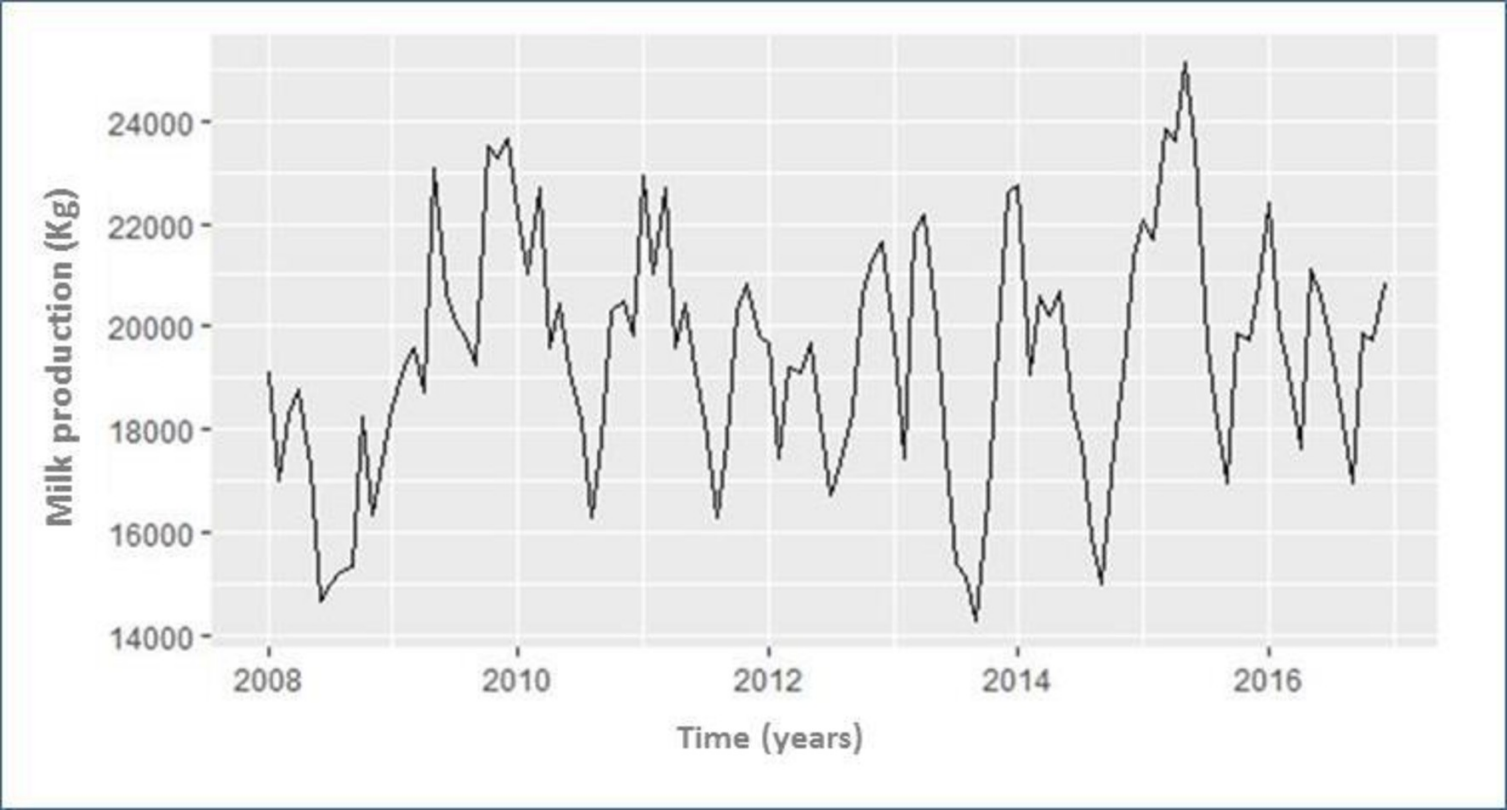
Time plot of milk production (kg) by year in dairy cattle maintained in the Andean highlands between January 2008 and December 2016. The horizontal trend, cycle, and distribution of milk production (kg) and spectral of the empirical fluctuation process (observational values) in the study period.

The whole 9-year period was considered as base series for the milk production records (Fig 3). The model, the R^2^estimation, and the R^2^ adj. were calculated. The R^2^ for the autoregressive linear model was <1% indicating that calendar month was a very poor predictor of the series. Over this period, the total number of dairy cows did not change substantially. However, upon decomposition of the actual data into trends, it was revealed that a decreasing trend in milk production could be observed over the period 2008–2014. Then, the milk production was gradually raised from 2014 to 2016 (Fig 3).

### Autocorrelation (ACF), partial autocorrelation (PACF) and analysis of residuals

**Fig 4:**
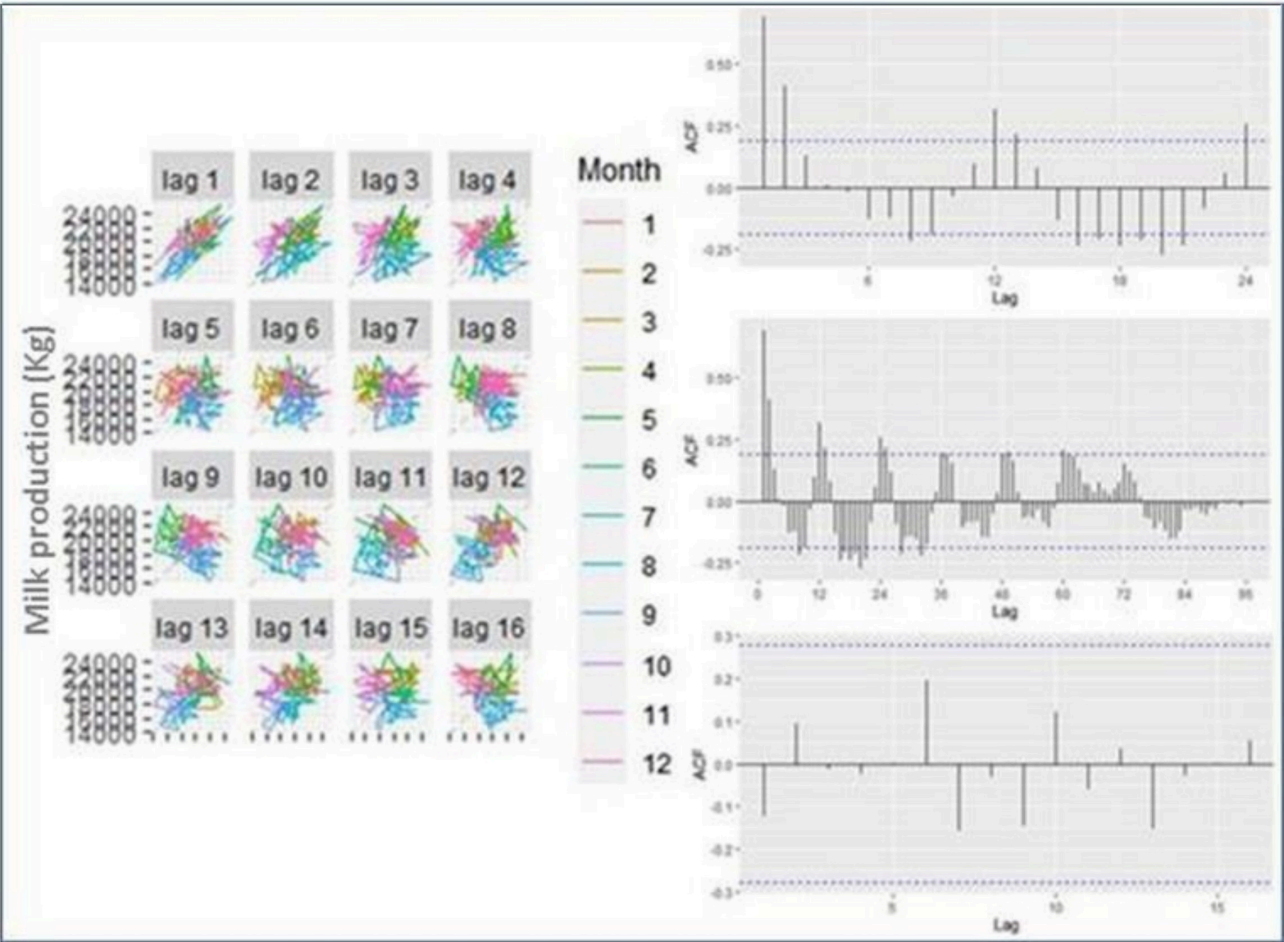
Autocorrelation (ACF) analysis of the linear relationship between lagged values of the time-series. Left: Scatterplots of milk production (kg) by month in dairy cows maintained in the Andean highlands where the horizontal axis shows lagged values of the time series. Each graph shows *y*_*t*_ plotted against *y*_*t−k*_ for different values of *k*. Right (top): ACF of a non-trended time-series tends to have positive and negative values that slowly decrease as the lags increase. Right (middle): ACF of a non-seasonal time-series showing that autocorrelations are not larger for the seasonal lags than for other lags. Right (bottom): ACF of a non-trended and non-seasonal time-series (combination of both effects).

The autocorrelation (ACF) and partial autocorrelation (PACF) indicated which model to use. The autocorrelation represented the key parameters of the SARIMA model (`p´ and `q´) more likely to be ≤2 (out of 95% CI). The results showed a non-seasonal configuration (ACF = 0.342, PACF = 0.265, both p < 0.05) indicating that SARIMA model (1, 0, 0) x (2, 0, 0)_12_ represented the most adequate model for milk production value estimation. Then, ACF and PACF showed no significantly non-zero autocorrelation in any lag, so the data were independent and demonstrates a good fit for the model. The analysis of residuals of the model in ACF and PACF were within the limit of the confidence interval (same for all lags). The residuals were randomly distributed in time. Various combinations, including different autocorrelation terms were analyzed (Fig 4).

The cross correlation between monthly values of the incidence of the season of the year (rainy/dry) for twelve lags in each year showed no differences between them, with a coefficient of 0.583 (95% CI: 0.325, 0.908) for rainy period and -0.492 (95% CI: -0.146, -0.672) for dry period. Lagged scatter-plots indicated serial dependence up until at least 12 months (Fig 4).

**Fig 5:**
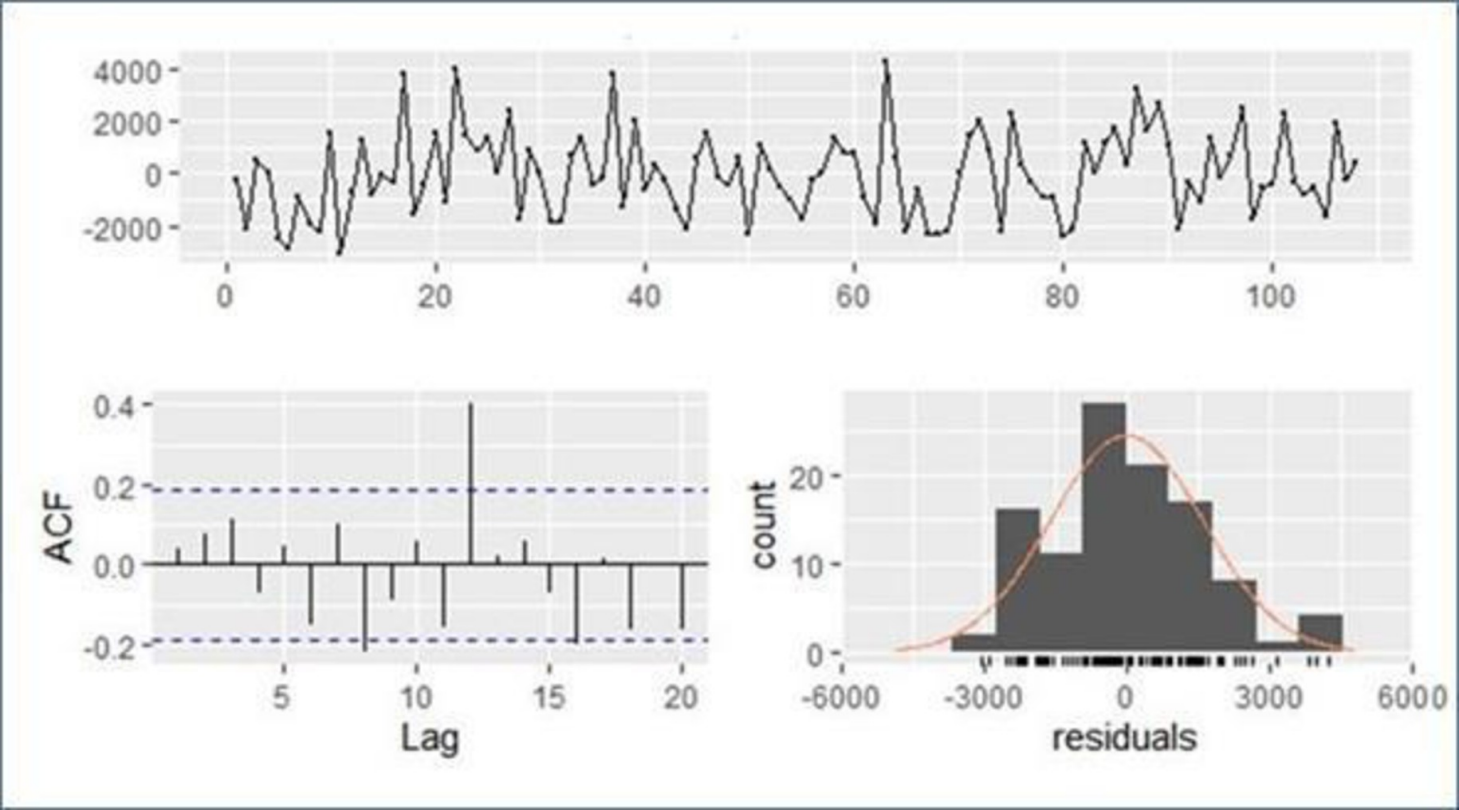
Residual diagnostic analysis for the SARIMA model. Different plots show residual fitted values to the monthly milk production (kg) data for future forecasting model. Top: Plot of residuals from forecasting the milk production (kg) by month in dairy cows maintained in the Andean highlands using the naive method. Bottom left: ACF of the residuals from the naive method applied to the milk production (kg) by month in dairy cows maintained in the Andean highlands. The lack of correlation suggests the forecasts are well performed. Bottom right: Histogram of the residuals from the naive method applied to the milk production (kg) by month in dairy cows maintained in the Andean highlands. The right tail seems a little too long for a normal distribution.

Hence, the residuals of all potential SARIMA models were not white-noise series (p<0.05), so the variables in the white-noise series were independent and identically distributed with a mean of zero (Fig 5).

Fig 5 verifies that the errors were concentrated in the range of (−0.23, 0.4), which means that there was no variance (errors around the mean). The residuals showed no linear correlation when tested by the ACF plot and the Ljung–Box test (χ2 = 42.031, df = 23, p > 0.05) and the Shapiro–Wilk test showed no residual normality (W = 0.5932, p < 0.05).

### Milk production forecasting using SARIMA model

**Fig 6:**
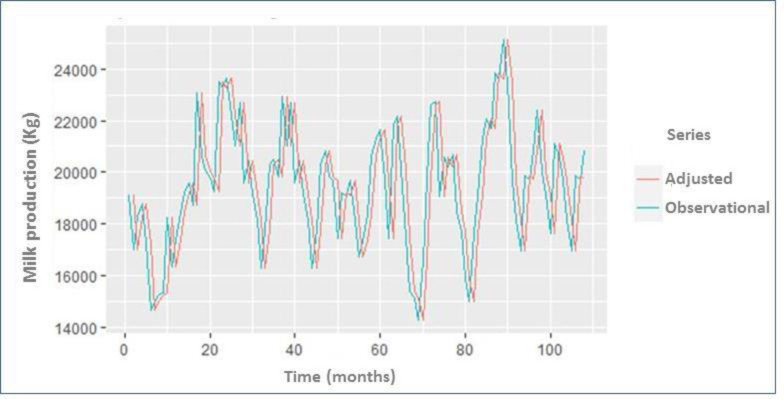
Comparison of monthly milk records and fitted SARIMA model for forecasting the milk production in dairy cows maintained in the Andean highlands. Plot shows the milk production over the 108-month period. The plot has been performed by using monthly data (observational and adjusted). The “observational” (light green) model showed the time plot using empiric data. The “adjusted” (red) fitted model showed the time plot after the data correction process.

After identifying the best fit model, the next step was to forecast milk production along time. The results of the fitted values (108 months) and the predicted milk production show that the forecast milk (kg) for both periods were within the allowable range and can therefore be justified in predicting future milk production. Comparison of monthly observational milk records (values predicted by the model and the observed values) and fitted SARIMA model between 2008 and 2016 are represented in Fig 6.

The model provided a seasonality pattern of milk production with low values for the dry season (about 65%) and high values for the rainy season (75%). For this reason, it was important to model these patterns at each season to obtain solid information detecting abnormal milk production events and support the decision-making. These oscillations of predicted productions were similar to observed values (Fig 6). Analysis of the difference between the observed and predicted values of milk production shows notable amplitudes in the whole frequency range (both shown typical white noise behavior). The differences and the Kolmogorov-Smirnov test indicate that this noise closely follows a normal distribution (Mean ± SD = -0.2 ± 1.4). Fig 6 shows the difference between the predicted and observed milk production with respect to the observed milk production (95% CI). The correlation between the difference and the observed milk production was 0.638 (p = 0.037), and the slope of the trend of the difference with respect to the observed milk production was 0.27 (p = 0.027). These results suggest that the model can adequately forecast the monthly milk production (Fig 6).

### Definition of the best-fitting SARIMA model

**Fig 7:**
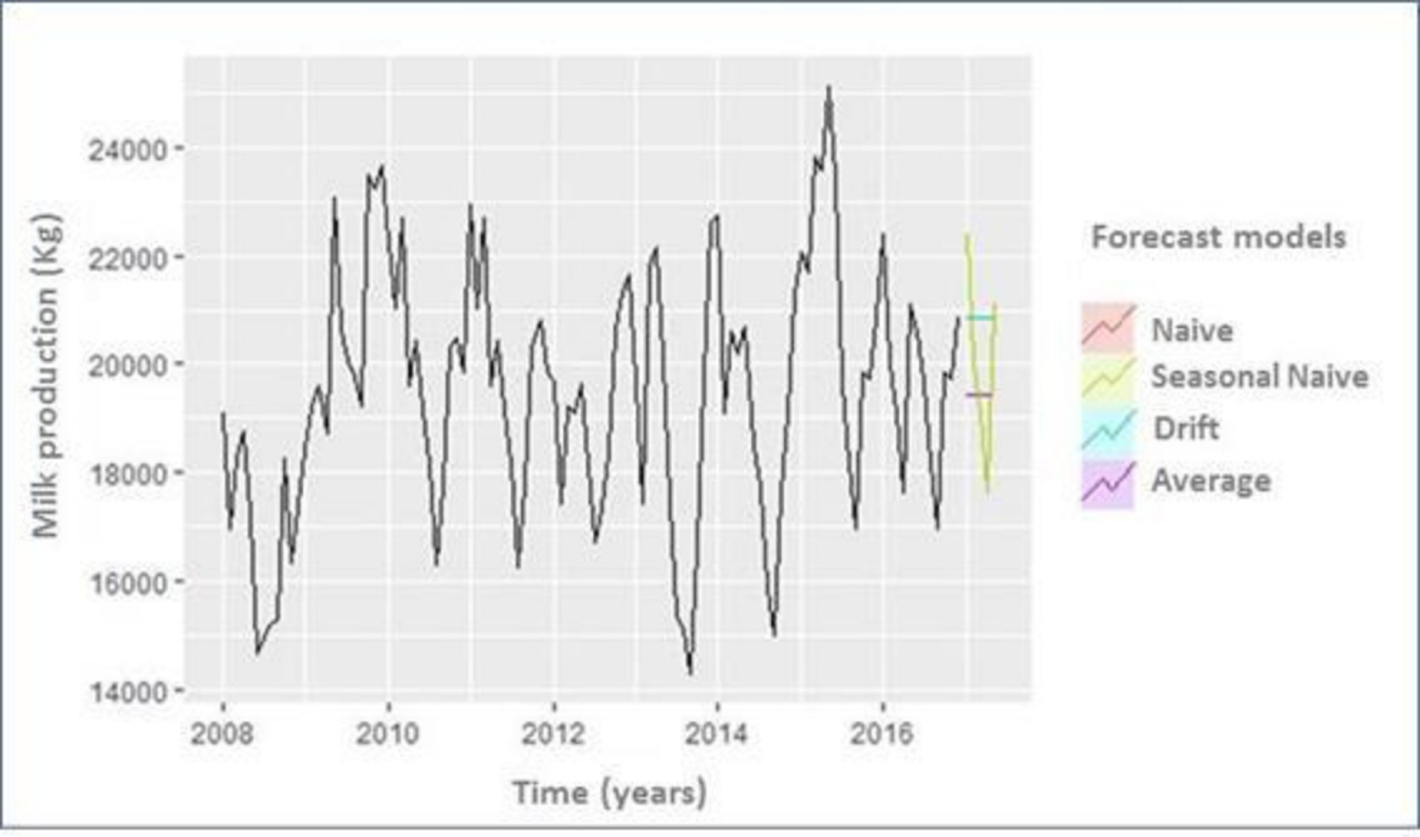
Forecasting the milk production in dairy cows maintained in the Andean highlands using SARIMA model from observational data obtained from 2008 to 2016. Plot shows the milk production over the 9-year period. The plot has been performed by using complementary-simple forecasting methods (naive, seasonal naive, drift, and average model). The "seasonally naive" forecast (light green) showed a very similar productions pattern of those of the past years, indicating that the known data were processed accurately. This fact leads to have an increased probability of obtaining an appropriate and efficient forecast.

The hypothesis of a non-stationary condition was tested by the Dickey Fuller test (p = 0.02811). An autocorrelation analysis of errors showed adequate adjustment (Portmanteau Q test; p > 0.05). The seasonal pattern of milk production was significantly explained by SARIMA model, with a delay of one month (p = 0.001). The milk production was significantly associated with the month of the year confirming the overall decreasing trend till 2014. Then, the model corresponded to a first-order moving average model indicating a weak dependence on random fluctuations from the previous years. The first step for forecasting milk production in dairy cows maintained in the Andean highlands using SARIMA model from observational data obtained from 2008 to 2016 is shown in Fig 7.

The serial milk production records show 12-month seasonal oscillations. Cross correlation of this term with the residual left in the rate by SARIMA (1, 0, 0) x (2, 0, 0)_12_ shows than none of lag coefficients was significant, therefore the environmental seasonal influence on milk production was determinant. Therefore, this model was defined as the final model with suitable parameters based on the minimum value according to the Akaike Information Criterion (AIC) (429.78).

**Fig 8:**
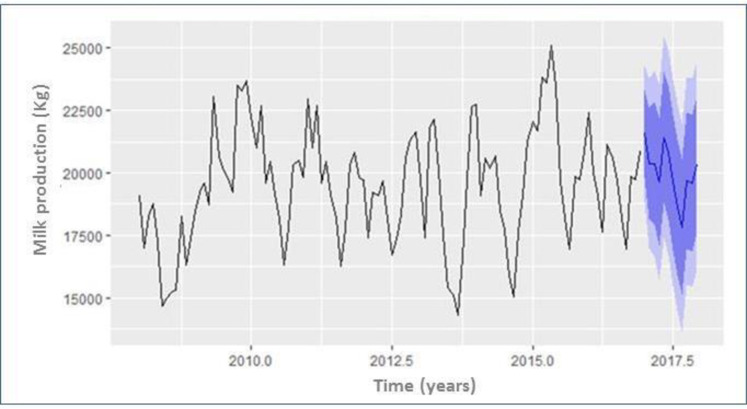
SARIMA model plot for forecasting the milk production in dairy cows maintained in the Andean highlands. The plot shows the milk production trend (observational data, black line) and the respective forecast (blue line) for the next 12 months (2017). A similar pattern is observed over the 9-year period showing high peaks in rainy-season months (October to March) and bottom peaks in dry-season months (April to September). The 80% confidence interval is shown in the middle (blue shaded area) while the 95% confidence interval is shown above and below (light blue shaded area).

This SARIMA (1, 0, 0) x (2, 0, 0)_12_ model was interpreted as follows: the number of lag observations included in the model or lag order was equal to one (p = 1), the degree of differences was equal to zero (d = 0), the order of moving average was equal to zero (q = 0), the last seasonally offset observation was used in the model (P = 2), seasonal differences were equal to zero (D = 0) and moving average order in seasonality was equal to zero (Q = 0), and yearly seasonal was set at 12 (m = 12). All values of the forecasted data were higher than the observed values. Although these results were not concurrent with the results of the evaluated model, the actual values were within the low and high limits at both the 80 and 95% significance level, so the SARIMA model can be used to forecast the milk production records (Fig 8). The predicted milk production values based on the best-fitted model case followed an analogous pattern of observational milk production values in 2008–2016 are shown in Table 1.

**Table 1:** Monthly predicted milk production values (2017) based on the best-fitted model obtained from the observational milk production (kg) in the 9-year study period (2008–2016).

Month	Forecasted milk production (kg)	Confidence Interval (CI)
January	15.14	[12.39–18.35]
February	16.75	[12.51–17.74]
March	15.54	[12.21–18.37]
April	14.58	[11.45–17.34]
May	12.42	[9.73–14.18]
June	12.82	[10.14–15.27]
July	12.29	[10.04–15.98]
August	12.92	[9.62–16.82]
September	13.94	[10.69–17.59]
October	15.15	[12.38–18.89]
November	15.43	[11.85–17.36]
December	15.83	[11.05–16.97]

The predicted milk production mean values for 2017 based on the best-fitted model case followed an analogous pattern of observational milk production values in 2008–2016. All values of the forecasted data were higher than the observed values. The actual values were within the low and high limits (80–95% significance level), so the SARIMA model can be successfully used to forecast the milk production in the following years.

After adding the autoregressive term and the term representing seasonal influence of the environmental variables, and adjusting by linear regression, the model acquires the final form: It = 0.438It-1, where It is the milk production for any month t, It-1 is the observed milk production in the preceding month. Mean Absolute Error (MAE), Root Mean Square Error (RMSE), and Mean Absolute Percent Error (MAPE) were used as selection criteria. This model explains a substantial percentage of the observed variability in milk production (R^2^ adj. = 76%, F = 23.81, df = 1, p < 0.0001). Quality indexes of the adjusted R^2^ adj. have shown the smallest values (MAE = 46.00; MAPE = 9.00; RMSE = 8.00) for a monthly model of environmental changes. The external validation showed the lowest MAPE values (17.80) and RMSE (59.63) for a monthly scale of seasonal changes indicating the model had a reasonable forecasting ability. Moreover, the predictive accuracy of the SARIMA model was assessed from the training and test data of the studied time-series with Theil’s U-statistic value equal to zero. Thus, the statistical model, using SARIMA showed a satisfactory goodness-of-fit and the model base rate residual in the form of white noise was normally distributed.

### Milk production forecasting comparison: SARIMA-ETS-HW

The ETS forecasting method was carried out following a standard procedure. For the ETS (error, trend, seasonality) approach, the ETS (A, N, N) was selected as the final model which could be interpreted as the model with non-damped trend (N), non-seasonality (N), and additive errors (A). After ETS model application the forecasting was carried out (Parameters: *θ*1 = *α* – 1; C.I.: 80–95%) with AIC: 437.92 and MAPE: 15.27. Similarly, the Holt-Winters (HW) model was performed following a standard procedure. This model falls into two categories: additive and multiplicative. In order to select the best model, fitted values for the additive and multiplicative models were analysed. Then, the fitted values of the models were compared to the original series. The model that best corresponds to the milk production values was selected to compare with the best SARIMA model. The intermediate best model using the selection criteria was the HW additive model. Overall, the SARIMA model (AIC: 429.78 and MAPE: 9.00) performed marginally better than the HW model (AIC: 418.49 and MAPE = 17.21). In particular, the model predicted well (out-of-sample) for certain periods including the forecast among years. An improved adjustment of the parameters for the SARIMA model allowed us to make better predictions for the increased production periods. Therefore, the SARIMA model approach had a better forecast accuracy compared with the ETS approach or the HW model. It is worth noting that these results are for out-of-sample predictions and that both models have similar MAPE values. Both models could be used to forecast milk production values; however, according to the selection criteria, the milk production values predicted by the SARIMA model were closer to the original milk production values than those predicted by the HW model. The actual and predicted values for milk production based on the validation dataset are shown in Figs 7 and 8. It was demonstrated that SARIMA model-derived values were notably closer to the actual values of milk production than those from other models. Interestingly, for the last 6-month period, the HW approach predicted milk production with an increased degree of accuracy whereas the ETS appeared to perform well in prediction over the course of 12-month period. The milk production presented annual seasonality with a quite stable trend over time. Interestingly, this pattern was consistent for all years. When the data was decomposed into a seasonal component, a seasonal pattern was clearly shown. A decreasing trend and seasonal pattern was observed during April to September, coinciding with the dry season. During December to March, in the rainy period of the year there was also an increase of milk production. The comparison of the performances obtained from the different forecasting methods performed in the study period (2008–2016) based on the milk production values are shown in Table 2.

**Table 2:** Comparison of the performances from the different forecasting methods performed in the study period (2008–2016) based in the milk production values.

Criteria	Milk production forecasting method		
	SARIMA	ETS	HW
AIC	429.78	437.92	418.49
MAPE (%)	9.00	15.27	17.21

According to Akaike Information Criterion (AIC) and Mean Absolute Percent Error (MAPE) values, the SARIMA (1, 0, 0) x (2, 0, 0)_12_ time-series model resulted slightly better forecasting model compared to others (although AIC value was similar in the three methods, MAPE value was much lower compared to those obtained in the other forecasting methods).

## Discussion

The forecast models designed and evaluated in the present study attempted to provide a simple tool to obtain reliable estimations of the expected milk production one year in the future based on the observed milk records during a 9-year period in the Andean highlands. To the best of our knowledge, this was the first study to forecast milk production using time-series forecast models in this area. This methodology was based on the hypothesis that the milk production in any particular time period could be used to forecast future milk yields mostly due to the environmental climate dependency. In the present study the forecast models worked well in the Andean highlands and allowed linking seasonal detected characteristics with the dairy production status and monitoring their temporal evolution; however, they have not yet proven in other highland regions. The different climate seasons were responsible for a large part of the variability in the milk production in this area; however, more complex factors could be involved to explain this variability but they were not yet investigated [28, 29]. This has led some researchers to develop statistical models in which milk production records were standardized in different conditions [30–40]. The models derived from the present study analyzed seasonal factors in the preceding month as factors predisposing milk production variability in the Andean highlands. The results showed the relation of the milk production with season in the preceding month. Several studies have found a correlation between the milk production and variations in climatic variables during several preceding months, or even with inter-annual variations [30, 32, 33]. The forecast models derived in the present study showed a strong correlation between the milk production and the seasonal month evaluated. An explanation for this finding could be that the rainy season (October to March) the pasture availability in the Andean highlands increased due to the increased precipitations during these rainy months as occurs in other similar environments [11, 13]. The situation may be different in other higher altitude and drier areas where variations in the temperature (<5°C) and precipitations (< 500 mm/year) could block the pasture growth and this fact may significantly affects forage availability in these areas [6]. Therefore, the extrapolation of the results obtained in this study should be considered only for specific regions sharing similar environmental characteristics where rainfall is a seasonal important factor [11, 12, 33].

In the present study the variable milk production has not been log transformed in the forecast models used. Log transformation has been carried out in other studies to avoid potential problems related to methods that assumed normality of data distribution [41, 42]. However, this log transformation procedure reduces the sensitivity and the transparency of the resulting models [42]. In this regard, it is important to have a historic stability during data collection [42]. In the present study the monthly milk production records from 2008 to 2016 remained constant, using the same measurement methods, the same dairy production system, the same management, and the same nutritional and environmental conditions. The observed decreasing trend estimated using the SARIMA statistical analysis regarding milk production records from 2008 to 2014 was unknown. There have been no further developments in the farm, neither as regards the number of lactating cows nor about milking routine, new management schemes or new staff recruitment. However, the most probably reason regarding the increasing trend in milk production from 2014 to 2016 could be linked to the presence of known factors such as cow replacement rates. The SARIMA (1, 0, 0) x (2, 0, 0)_12_ time-series model performed better than the HW or the ETS model; however, the difference among models was marginal. The model indicates that the mean of milk production records did not show significant change over time. The main advantage of these models was the consideration of seasonal differences which can be useful to predict milk production [30, 32, 33]. However, their implementation requires appropriate datasets as those compiled by farm record systems which are currently scarce for livestock in the Andean highlands. The presence of stable seasonality is important and lends itself well to forecasting perspectives that decompose the milk record time-series into seasonally adjusted components to identify environmental predictors of exposure [33]. All time-series follow the same pattern where the present milk records depend mainly on the moving averages of the previous month and season [33]. It is widely accepted that each method has a different capacity for dealing with data related to seasonal patterns removing the derived effects form noisy data, excluding the data-related cyclical/seasonal components, and forecasting future values of the series [43]. Hence, the SARIMA model use to have difficulties in detecting the non-linear data patterns compared to other time-series models such as ETS [44]. Forecasting monthly records has crucial implications for dairy producers to formulate a strategic plan organizing future milk production [33]. An accurate forecast could allow avoiding drastic drops in milk production and perform a better nutritional planning scheme during the dry season months when pasture production drops [11, 45]. Cross correlation between time-series of milk production was high when lag was zero. This fact indicates that changes are synchronic. Correlations between seasonal indices also indicated that milk production followed a similar seasonal pattern that is generally defined by a higher milk production during rainy season than during dry season. These results suggest that milk production is bound to temporal factors. These monthly autocorrelations can be explained by the pluviometric fluctuations and the pasture growth depending on the season of the year considered [11, 45]. The SARIMA time-series model performed slightly better values of MAE, MAPE, and RMSE which were relatively low for milk production records compared to other forecast models [36, 46]. Adjusted models have properly captured the trend of milk production records and they predict future records with high precision as they met the criterion of the absence of residual autocorrelation [47]. Despite the Shapiro–Wilk test shows the absence of normal residuals was not a necessary precondition in the diagnosis of ARIMA models the predictive capacity of the model was not affected by this fact [48]. However, the identification step has been the most critical stage of developing the ARIMA model [48]. The relatively medium-high RMSEs could be due to the small number of animals of the population monthly tested. On the other hand, factors related to herd management were not considered in the models because, as stated before, no changes related to this factor were observed over the 9-year period. While SARIMA model tend to overestimate the forecasts the comparisons across the models were still solid [33, 44]. In any case, the SARIMA model showed great capability to capture irregular fluctuations and seasonality patterns [33]. Furthermore, the SARIMA performed well in dealing with the data autocorrelation, trend, and irregular patterns to deliver more accurate forecasts [32, 33]. The Dickey-Fuller test was performed to determine seasonal trends and the Portmanteau test was performed to define white noise in the model residuals. Hereafter, the seasonal pattern of milk production was significantly explained by time-series models and observed values were adequately predicted. As stated before the best forecasting model based on the accuracy included the SARIMA model; however, SARIMA model was relatively more difficult to fit in terms of the specification of the milk records and the relatively longer time taken to run the model. On the other hand, the error parameters have shown the smallest values for a monthly model of milk production changes. By comparing predicted to observed data, those records of dairy production that exceeded the upper limits of a conventional 95% predicted interval were identified as milk peaks. In addition, the autocorrelation functions (ACF) and partial autocorrelation plots (PACF) were performed to determine model parameters and the Akaike Information Criteria (AIC) were employed to select the best-fitted model. In any case, these time-series models also include trend and seasonality for describing profiles of monthly production and detecting productive variability. Moreover, it is possible to forecast for periods longer than a month; however, the predictions may inherently not be as accurate as observed previous studies [33, 44]. Our results demonstrated that SARIMA method was adequate and improved the accuracy forecast of the 2017 validation data. Moreover, based on SARIMA model results the milk production during dry months (April to September) was lower compared to the rainy months (December to March) due to the climatic seasonality [49]. Thus, milk marketing should be planned to manage these seasonal variations related to dairy production activity to create a production control system by reducing both point and prediction interval errors [50]. Therefore, management strategies could be crucial for reducing economic losses in dairy herds, specifically in the South American regions where the food resources are scarce [51, 52]. This is particularly important given the lack of accurate monitoring systems in similar highland regions [53]. Future studies will continuously update the forecast model developed in the present study and compare it to other emerging models over time to detect model failures and make pertinent improvements. Although the arising productive and economic consequences have never been formally assessed, additional research would be useful to understand the consequences of seasonal milk production decrease in the region, as well as to provide better control programs increasing the effectiveness of animal production systems [7, 8]. These findings have direct and practical applications for both farm-level and national-level dairy production which potentially could result in more cost-effective economic strategies applied in the Andean highlands.

## Conclusions

In conclusion, the milk production in the Andean highlands displayed a cyclical pattern of milk records that seems to be repeated every single year. The periods of lower productivity (between April and September) indicated that March was the critical month because of the need of starting the nutritional supplementation program of the herds. More research is necessary to determine the dairy production cycle in other Andean regions mainly on factors inherent to specific animal production systems correlating them with the pattern of milk production in these areas. Finally, the SARIMA predictive model was the most adequate to predict from the developed time-series demonstrating to be a useful tool in decision-making and planning economic strategies in dairy cattle where cyclical seasonal climatic changes occur every year. These findings have practical applications and the approach could be adapted to other data related to animal production systems or even economic fluctuations in similar geographical Andean regions sharing analogous environmental characteristics.

### Limitations of the study

The main limitations of the study are based on the following factors; first; milk production systems are not following a standardized methodology in the Andean Highlands which is a frequent characteristic due to the nature and culture of the inhabitants in this region. Second, lack of modern technologies for milk production control (e.g. milk measuring devices, milk quantity and quality control, udder hygiene assessment, etc…). Third; lack of control of diseases that reduce the milk production such as clinical and subclinical mastitis. Forth; lack of a standardized nutritional scheme to feed animals avoiding milk production losses. All these combined factors have been shown to be contributing to underestimating the milk productivity in the Andean highlands. However, the above-mentioned underestimations are ubiquitous in the Andean region. Thus, the effects of those potential underestimations may be having minimal effect.

## Abbreviations

ARIMA: Auto regressive integrated moving average
SARIMA: Seasonal auto regressive integrated moving average
AR: Auto regressive
MA: Moving average
MAE: Mean absolute error
RMSE: Root mean square error
MAPE: Mean absolute percentage error
ADF: Augmented Dickey- Fuller
AIC: Akaike information criterion
AICc: Akaike information criterion corrected
MLE: Maximum likelihood estimation
ACFs: Autocorrelation functions
PACFs: Partial autocorrelation functions
STL: Seasonal and trend decomposition using Loess
ETS: Error, Trend, Seasonality
HW: Holt-Winters
